# The relationship between claimed restorations and future restorations in children and adolescents: An observational follow-up study on risk categories for dental caries

**DOI:** 10.1371/journal.pone.0259495

**Published:** 2021-11-12

**Authors:** Riët Hummel, Wil van der Sanden, Josef Bruers, Geert van der Heijden

**Affiliations:** 1 Department of Oral Public Health (OPH), Academic Centre for Dentistry Amsterdam (ACTA), University of Amsterdam and Vrije Universiteit Amsterdam, Amsterdam, The Netherlands; 2 Zilveren Kruis Achmea, Zeist, The Netherlands; 3 Department of Dentistry, Quality and Safety of Oral Healthcare, Radboud Institute for Health Sciences, Radboud University Medical Center, Nijmegen, The Netherlands; 4 KNMT, Royal Dutch Dental Association, Utrecht, The Netherlands; Universitat Bern, SWITZERLAND

## Abstract

Various models are available to assess caries risk in individuals. In general past caries experience is considered as the best single predictor for future caries development in populations. Likewise, recent restorations have been used to predict future restorations. We aimed to evaluate a classification model for risk categories for dental caries in children based on claims data from Dutch healthcare insurance company Zilveren Kruis. The baseline caries risk categories were derived from the number of claimed restorations in two baseline years (2010 through 2011). These categories were defined as low (no new restorations), moderate (1 new restoration), and high (2 or more new restorations). First, we analyzed the relationship between baseline caries risk categories and the number of new restorations during 3 years of follow-up (2012 through 2014). Secondly, we used negative binominal two-level analyses to determine the accuracy of our classification model in predicting new restorations during follow-up. Thirdly, we reclassified the participants after 3 years and determined the changes in the categorization. We included insurance claims data for the oral healthcare services in 28,305 children and adolescents from 334 dental practices for the period 2010–2014. At baseline, 68% of the participants were in risk category low, 13% in moderate and 19% in high. The mean number of new restorations during follow-up was 0.81 (SD 1.72) in baseline risk category low, 1.61 (SD 2.35) in moderate, and 2.65 (SD 3.32) in high. The accuracy of the multivariate model for predicting 0/>0 restorations was 50%. After 3 years, 60% of the study participants were in the same risk category, 20% were in a lower, and 21% in a higher risk category. Risk categories based on claimed restorations were related to the number of new restorations in groups. As such, they could support planning and evaluation of oral healthcare services.

## Introduction

Various models are available to assess caries risk in individuals aiming to predict future caries development [1, 2]. However, accurate assessment of caries risk is complicated due to the multifactorial etiology of dental caries and the complex relationships and interactions between its risk factors [3].

The available models vary in the number of included predictors (1 to 25); the kind of predictors (besides caries experience for example sociodemographic, behavioral, environmental, and biological); and the target population. To date, evidence that including a large number of risk factors results in more accurate predictions is lacking [1]. Moreover, past caries experience has been reported to be the most powerful predictor for future caries development [2], especially in populations [4]. As such, under the assumption of absence of overtreatment or undertreatment, past restorations might also be predictive for future restorations.

If the caries risk could be accurately predicted, a population could be categorized into risk categories for dental caries. This would have several advantages from a public health perspective. It could be used for planning and evaluation of oral healthcare services, as it could serve in planning of preventive measures in higher risk groups in order to prevent for future restorative treatment [2]; and in planning of the required capacity of oral healthcare providers. It could also be used to categorize a population to support fees in a risk-based capitation system [5]. And finally, a prediction model would allow for using restorations provided in a population as an outcome measure [6], and as such add value to the oral healthcare system.

Modeling of caries risk based on the actual and recent numbers of active carious lesions has been proposed by Mettes et al. [7]. This approach to caries risk assessment was modified and tested by Hummel et al. [6], using the number of claimed restorations as a surrogate for recent caries experience and hence as a proxy measure for the oral health.

In this study, we aimed to evaluate a classification model for risk categories for dental caries in children and adolescents based on claims data from Dutch healthcare insurance company Zilveren Kruis. Our purpose was to answer the questions: i) What is the relationship between predetermined caries risk categories and the number of new restorations over a follow-up period of three years?; ii) What is the accuracy of our classification model before and after multivariate modeling in predicting the number of new restorations during follow-up?; iii) What are the changes in the categorization after three years?; and iv) Were these changes different for groups with different demographics at baseline?

## Methods

Traditionally, children and adolescents in the Netherlands visit their dental practice (DP) once or twice a year for routine oral health examinations (ROEs) and, when considered necessary, preventive and restorative treatments. These dental procedures are claimed per item and their costs are covered up to 18 years old by a legally compulsory standard healthcare insurance. We used claims data from a healthcare insurance company as a proxy for the oral situation. The data were provided by Zilveren Kruis, a large healthcare insurance company in the Netherlands insuring over 3 million persons.

### Participants

Our participants were children between 0 and 12 years old in January 2010. Zilveren Kruis provided a dataset of children who met three criteria: as the study period was from 2010 through 2014, they had to be born between 1 January 1997 and 1 January 2010; they had to be insured by Zilveren Kruis for the entire study period; and had to have had claims for oral healthcare services. From this population, we excluded individuals who did not have at least one ROE per year during the study period to be able to analyze complete cases and to ensure we did not miss any indicated restorative treatments. Then, we excluded individuals with any inexplicable number of treatments. As the payment system in the Netherlands is fee-per-item, claims consisted of a code for the care procedure and the number of this procedure. In some cases, the number of procedures was zero or negative due to corrections in the financial administration. The data of these individuals were not reliable. Next, we excluded individuals who visited multiple DPs as we wanted to carry out multilevel analyses to correct for DPs. Multiple DPs were identified for each individual by the number of DPs that had claimed ROEs during the study period. Finally, we excluded individuals from DPs with less than 45 children to ensure enough accuracy for the multilevel analysis. We had claims records for 292,165 individuals from 3,294 different DPs. They claimed for a number of individuals ranging from 1 to 1,619. The 10% DPs with the highest numbers of individuals had claims for at least 45 children.

The selection process resulted in analysis of 10% of the sample in the available data. Compared to all children in the available data, the participants were older (mean age 7.2 (SD 3.0) versus 6.1 (SD 3.7), of lower socioeconomic status, and more often patient in a JTV (13% versus 4%). There were no differences in gender. A JTV is a center specialized in pediatric oral health care. Most of the JTVs are located in urban areas and provide extra attention to children from vulnerable groups.

### Assessment of risk categories for dental caries

Mettes et al. [7] described 4 predefined risk categories for dental caries and used them as part of risk-based clinical vignettes. These were developed for educational reasons in the Netherlands and aimed to support decision-making on recall intervals. The risk categories were mainly based on recent caries activity (newly developed or progressing carious lesions, or recent restorations, or both). We modified this caries risk classification into a model based on recent restorations, as this was the only recent caries activity that was visible in claims data. Our assessment period was set on 2 years, as it takes time for active carious lesions to progress towards a stage needing restorative treatment.

From there, our risk categories for dental caries were defined as:

**High**—two or more new restorations in the last 2 years;
**Moderate**–one new restoration in the last 2 years;
**Low**–no new restorations in the last 2 years.

A description of the risk categories from Mettes et al. and the translation into our risk categories are provided in (S1 Table).

### Data collection

Zilveren Kruis provided a dataset from their healthcare insurance reimbursement registry in February 2016. This dataset consisted of claims data for the period January 2010 through December 2014. They provided the data in compliance with their regulations for data safety and privacy protection. The shared data could not be traced back to individual participants, and included an identification number, the year and month of birth, gender, the socioeconomic status and claims for provided oral healthcare services. These claims could concern ROEs, intra-oral radiographs, professional fluoride applications, professional toothcleaning, dietary analyses, plaque scores, sealants, restorations, prefabricated crowns for primary teeth, tooth extractions, and endodontic treatments. Information per claim was the number of the procedure (for example in case of extraction of 2 teeth, the procedure was tooth extraction and the number of procedures 2), an (not traceable) identification number of the DP that provided the care, and whether this was a general DP or JTV. There was no information on the tooth number of treated teeth. This information was only available in the second half of the study period as it was since then mandatory to add the tooth number to claims. An overview of the provided information per participant can be found in Table 1.

**Table 1:** Information per participant.

Data were extracted from healthcare insurance company Zilveren Kruis for the period January 2010 through December 2014
Characteristics of the participant
• (Not identifiable) identification number of the participant
• Year and month of birth
• Gender
• Socioeconomic status (based on postal code home address)
Claims for
• Routine oral health examinations
• Intra-oral radiographs
• Professional fluoride applications
• Professional toothcleaning
• Dietary analyses
• Plaque scores
• Sealants
• Restorations
• Prefabricated crowns for primary teeth
• Tooth extractions
• Endodontic treatments
Information per claim
• (Not identifiable) identification number of the dental practice
• Treated in JTV (yes/no) ^a^
• Number of procedures ^b^

^a^ JTV is a center specialized in pediatric oral healthcare

^b^ For example: two one-surface restorations will be claimed with care procedure code V11 and number of procedures 2

### Data processing

Year and month of birth were recalculated into age in January 2010 and subsequently divided into age categories (0–3 years old; 4–9 years old; 10–12 years old). These age categories were a proxy for having a primary, mixed or permanent dentition during the baseline years (2010 and 2011). The socioeconomic statuses (categories low, middle, high, unknown) were provided by Zilveren Kruis and were based on the postal codes of the home addresses of the participants. A table with figures per postal code area (neighborhood) from Statistics Netherlands was used for the categorization [8]. Zilveren Kruis derived the socioeconomic status from the average income per inhabitant.

Our analytic model is based on baseline data from a two-year period. Therefore, we summed the number of claims in 2010 and 2011 per procedure type. ROEs were dichotomized into 2 to 4 ROEs and more than 4 ROEs during baseline. We dichotomized intra-oral radiographs, professional fluoride applications, professional toothcleaning, dietary analyses, plaque scores, sealants, tooth extractions, endodontic treatment, and endodontic treatments in primary teeth into provided during baseline yes or no.

The number of restorations was counted by summing the number of claimed restorations and prefabricated crowns. This was used to determine the risk categories for dental caries according to the method described earlier. The risk categories at baseline were based on claims in the years 2010 and 2011, and those at follow-up on the years 2013 and 2014.

### Data analyses

The relationship between the baseline risk category for dental caries and the total number of restorations in the three years of follow-up was analyzed using a multilevel approach to the data analysis with two levels (DPs and patients). STATA (version 14.1) was used for analyzing the data. The -2 log likelihood showed that a random intercept for DP was necessary. We performed negative binominal multilevel analyses as the number of restorations was a count variable and the variance (10.96) was much higher than the mean (1.27).

Then, we derived a prediction model with a forward procedure using a cut-off point for the p-value of 5% to analyze which baseline variables most accurately predicted future restorations. The accuracy of this model was determined by analyzing the percentages in which the prediction model correctly estimated, overestimated or underestimated the number of restorations at follow-up, and by calculating the positive predictive value, negative predictive value and accuracy for the prediction of 0 or >0 restorations.

After 3 years, we reclassified the participants. The changes in participants’ risk categories were analyzed by comparing their risk category at baseline to that at follow-up. We described the percentages of participants in the same, in a lower, or in a higher risk category at follow-up. Subsequently, we did the same for various sociodemographic groups. These groups were based on age (0–3, 4–9, 10–12 years old in January 2010); gender (boys, girls); and socioeconomic status (low, middle, high, unknown). We calculated the differences between the percentages of the participants from the various sociodemographic groups and the percentages of all participants. Eventually, we did the same for participants with improved or deteriorated classifications. SPSS (version 26) was used for the descriptive statistics.

## Results

### Participants

**Fig 1:**
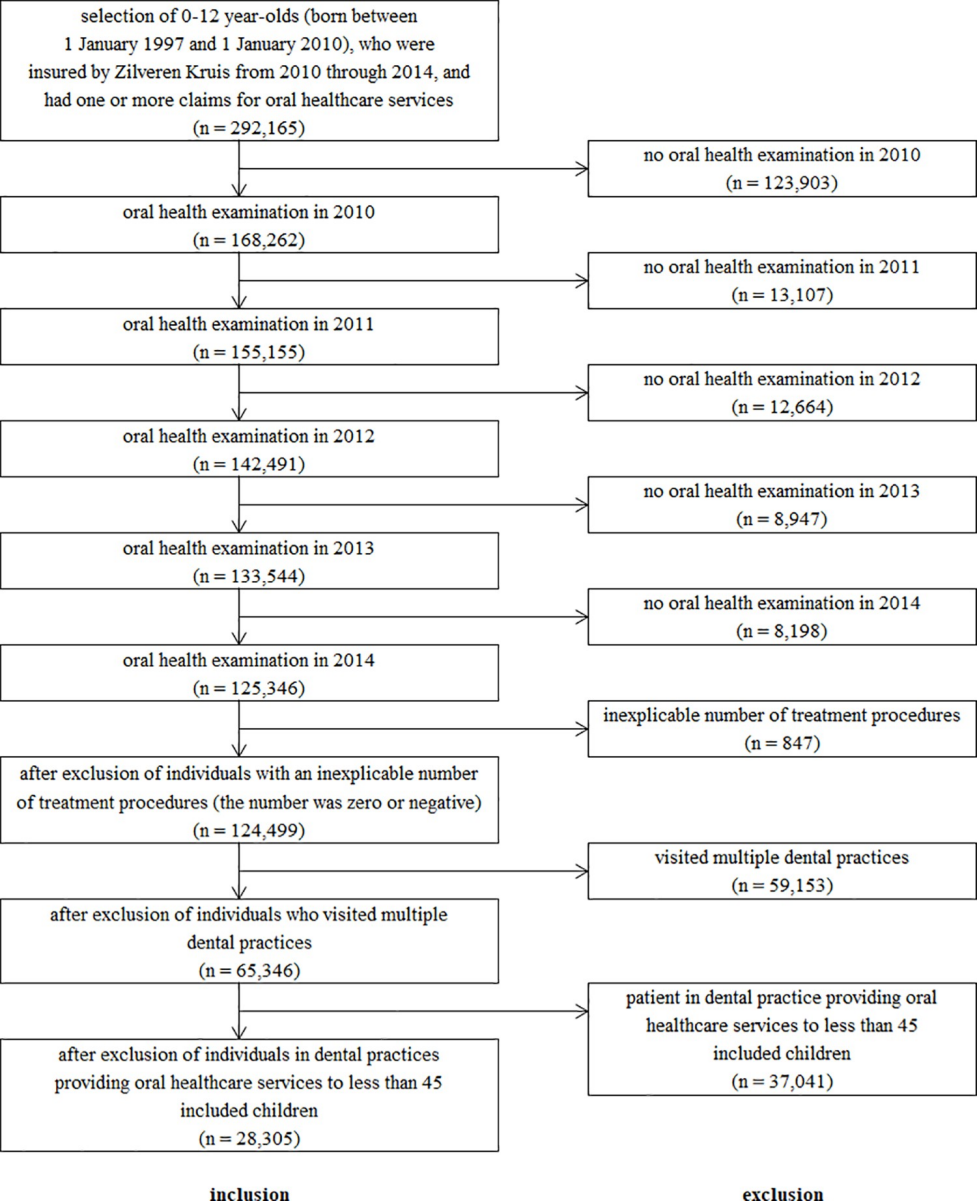
Flow diagram of the selection of the study participants.

The dataset from Zilveren Kruis comprised data of 292,165 children who met the initial selection criteria. From there, we excluded 166,819 individuals for not having had a yearly ROE and another 847 for having any negative care procedure number or any number of zero. Then, we excluded 59,153 individuals that had claims records from multiple DPs. And finally, we excluded another 37,041 individuals visiting DPs with less than 45 children. This left 28,305 participants from 334 different DPs who validly qualified for inclusion in this study. The flow diagram of the selection of the participants is provided in Fig 1.

### Risk categories for dental caries

At baseline, 68% of the participants was in risk category low, 13% in moderate and 19% in high. The characteristics of the study participants and the received oral healthcare services per risk category are provided in Table 2.

**Table 2:** Description of the variables per risk category for dental caries.

	Risk category for dental caries			
N (%)	low	moderate	high	all
	19,150 (67.7%)	3,650 (12.9%)	5,505 (19.4%)	28,305 (100%)
Age (January 2010)				
Mean (SD)	7.10 (3.15)	7.71 (2.64)	7.22 (2.66)	7.20 (3.01)
Category (January 2010)				
10–12	27.8%	28.1%	22.7%	26.8%
4–9	55.8%	65.6%	70.3%	59.9%
0–3	16.5%	6.3%	7.0%	13.3%
Gender				
Boy	51.1%	50.6%	51.1%	51.1%
Girl	48.9%	49.4%	48.9%	48.9%
Socioeconomic status				
High	28.8%	23.9%	20.8%	26.6%
Middle	40.6%	39.3%	37.1%	39.8%
Low	29.2%	35.8%	40.8%	32.3%
Unknown	1.4%	0.9%	1.3%	1.3%
Patient in JTV ^a^	12.1%	15.2%	15.8%	13.2%
More than 4 routine oral health examinations during BL ^b^	1.2%	2.2%	3.3%	1.7%
Intra-oral radiographs during baseline	22.1%	36.1%	43.2%	28.0%
Professional fluoride applications during BL	61.4%	74.3%	74.6%	65.6%
Professional toothcleaning during BL	41.7%	47.0%	47.4%	43.5%
Dietary advice during BL	0.1%	0.3%	0.6%	0.2%
Plaque scores during BL	13.2%	16.4%	20.5%	15.0%
Sealants during BL	31.0%	43.0%	48.0%	35.9%
Tooth extractions during BL	11.8%	20.0%	25.9%	15.6%
Endodontic treatments during BL	0%	0.8%	1.7%	0.4%
Endondontic treatments in primary teeth during BL	0%	0.8%	1.9%	0.5%
Restorations during BL	0%	100%	100%	32.3%
Restorations during FU ^c^	32.2%	55.4%	70.3%	42.6%

^a^ JTV is a center specialized in pediatric oral healthcare

^b^ BL is baseline (claims in 2010 and / or in 2011)

^c^ FU is follow-up (claims in 2012, in 2013 and / or in 2014)

### Relationship between risk categories and number of restorations

**Fig 2:**
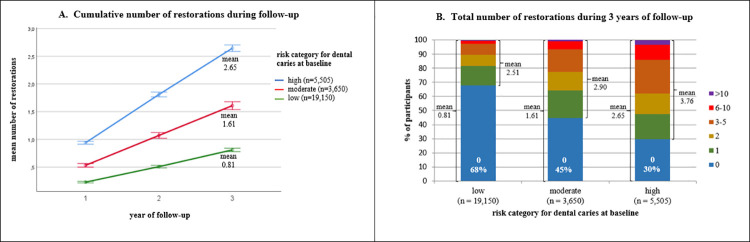
Number of restorations during follow-up. (A) Cumulative mean number of new restorations after 1, 2 and 3 years of follow-up per risk category for dental caries with error bars for the 95% confidence interval. (B) Distribution of the total number of new restorations per participant during 3 years of follow-up per risk category for dental caries at baseline.

Fig 2A shows the cumulative mean number of restorations after 1, 2 and 3 years of follow-up per baseline risk category for dental caries. It demonstrates that the increments were linear, and that higher risk categories at baseline preceded higher increments in the cumulative number of restorations. After 3 years of follow-up, the mean number of new restorations was 0.81 (SD 1.72) in risk category low, 1.61 (SD 2.35) in moderate, and 2.65 (SD 3.32) in high. Fig 2B provides the distribution of the total number of restorations per participant during follow-up. This shows that the larger means in higher risk categories were caused by both larger percentages of participants with restorations during follow-up and larger numbers of restorations per participant.

The accuracy of the predicted number of restorations based on the mean number of new restorations observed per baseline risk category rounded to the nearest integer, is described in Table 3A. Baseline risk categories correctly predicted the number of new restorations in 13% of all cases, and the number of new restorations ±1 in 72% of all cases.

**Table 3:** Accuracy of the predicted number of restorations at follow-up.

Percentage of participants with an underestimated, correctly estimated, or overestimated number of restorations during follow-up				
	risk category for dental caries at baseline			total
	low	moderate	high	
	n = 19,150	n = 3,650	n = 5,505	n = 28,305
** 3A Uncorrected** ^**a**^				
predicted > observed (over estimation)	68	64	62	66
predicted = observed (correct estimation)	14	13	11	13
predicted < observed (under estimation)	18	23	27	21
predicted = observed +/- 1	89	40	33	72
** 3B Prediction model** ^**b**^				
predicted > observed	54	55	54	54
predicted = observed	26	18	14	23
predicted < observed	20	27	32	23
predicted = observed +/- 1	87	62	44	75
** Accuracy of the prediction model**				
positive predictive value ^c^	35	55	70	46
negative predictive value	79	100 ^d^	0 ^e^	79
accuracy	43	55	70	50

^a^ Predicted was based on the average number of restorations during follow-up per baseline risk category for dental caries (low rounded 1; moderate rounded 2; high rounded 3)

^b^ Corrected for: age category, socioeconomic status, more than 4 routine oral health examinations during baseline, professional fluoride applications during baseline, professional tooth cleaning during baseline, plaque scores during baseline, extractions during baseline, endodontic treatments during baseline

^c^ The threshold was >0 restorations during follow-up

^d^ n = 2

^e^ n = 0

### Multilevel analysis and prediction model

The univariate results of the relationship between the various variables and the number of restorations during follow-up are described in (S2 Table). The results of the multilevel analyses of the relationship between the baseline risk categories for dental caries and the number of restorations at follow-up with different corrections are summarized in Table 4. The output of the final multilevel prediction model is shown in Table 5. Finally, the accuracy of the predicted number of restorations during follow-up is shown in Table 3B. Independent variables in the multivariate modeling for the number of restorations during follow-up were baseline risk categories for dental caries, age, socioeconomic status, and 6 procedures during baseline: professional fluoride applications, professional toothcleanings, plaque scores, tooth extractions, endodontic treatments, and more than 4 ROEs. The final prediction model correctly estimated the number of restorations in 23% of all cases. If we allowed a range of 1 for the outcome, this was 75%. The overall positive predictive value for >0 restorations was 46%; being 35% in risk category low, 55% in moderate, and 70% in high. The overall negative predictive value for 0 restorations was 79%. This was all accounted for by risk category low. The overall accuracy was 50%; it was 43% in risk category low, 55% in moderate, and 70% in risk category high.

**Table 4:** The uncorrected and corrected number of restorations during follow-up per baseline risk category for dental caries.

Number of restorations during follow-up (95% confidence interval)			
	Risk category for dental caries at baseline		
	low	moderate	high
uncorrected	0.81 (0.79–0.83)	1.61 (1.52–1.70)	2.65 (2.54–2.76)
corrected ^a^	0.75 (0.72–0.79)	1.48 (1.39–1.58)	2.31 (2.19–2.45)
corrected ^b^	1.17 (1.08–1.26)	2.39 (2.18–2.61)	3.75 (3.44–4.08)
corrected^c^	1.16 (1.08–1.25)	2.37 (2.17–2.59)	3.68 (3.38–4.00)

^a^ Corrected for dental practice

^b^ Corrected for dental practice, age category, gender and socioeconomic status

^c^ Corrected for: age category, socioeconomic status, more than 4 routine oral health examinations during baseline, professional fluoride applications during baseline, professional tooth cleaning during baseline, plaque scores during baseline, endodontic treatments during baseline, extractions during baseline (final prediction model)

**Table 5:** Results of the final negative binominal multilevel forward prediction model (cut-off point 0.05) for the prediction of the number of new restorations during follow-up and the influence of covariates.

		Reference	RR ^a^	P-value	95% CI ^b^
Constant			1.16	<0.001	1.08–1.25
Baseline risk category	high	low	3.17	<0.001	3.02–3.33
	moderate	low	2.04	<0.001	1.92–2.16
Age category	≥10	0–3	1.11	0.01	1.03–1.21
	4–9	0–3	0.70	<0.001	0.65–0.75
Socioeconomic status	high	low	0.74	<0.001	0.69–0.78
	middle	low	0.83	<0.001	0.79–0.88
	unknown	low	0.80	0.02	0.67–0.96
More than 4 routine oral health examinations during BL ^c^	yes	no	1.18	0.03	1.02–1.37
Professional fluoride applications during BL	yes	no	0.84	<0.001	0.79–0.89
Professional toothcleaning during BL	yes	no	0.89	<0.001	0.84–0.93
Plaque scores during BL	yes	no	1.12	0.001	1.05–1.20
Tooth extractions during BL	yes	no	1.10	0.001	1.04–1.16
Endodontic treatments during BL	yes	no	1.32	0.04	1.02–1.73

The model is corrected for dental practice

^a^ Rate ratio. The corrected number of new restorations for baseline risk category moderate is 2.04 * 1.16 = 2.37; and for high 3.17 * 1.16 = 3.68.

^b^ Confidence interval

^c^ BL is baseline (claims in 2010 through 2011)

### Changes in risk categories for dental caries

Table 6 shows the percentages of participants in the same risk category after 1, 2 and 3 years. Table 7 describes the changes in the categorization after 3 years for all participants and participants from different sociodemographic groups. After 3 years of follow-up, 60% of all study participants were in the same risk category for dental caries, 20% moved to a lower risk category, and 21% to a higher risk category. Risk category low was the most stable category. Three quarters (74%) of the participants in risk category low at baseline were also in this risk category at follow-up. Risk category moderate was the most unstable one. After 3 years, only 18% of the participants remained in this risk category. Most participants changed from moderate to low risk (57%), and a quarter (25%) changed to high. From the participants in risk category high at baseline, over one third (38%) was still in high, so, 63% was in a lower risk category at follow-up.

**Table 6:** Percentages of participants in the same risk category after 1, 2 and 3 years.

		The percentages of participants in the same risk category		
risk category for dental caries at baseline	at baseline	after 1 year	after 2 years	after 3 years
*based on*	*2010 + 2011*	*2011+2012*	*2012+2013*	*2013+2014*
high	100	66	41	38
moderate	100	44	20	18
low	100	87	76	74

**Table 7:** Changes in risk categories for dental caries after 3 years of follow-up for all participants and participants from different sociodemographic groups.

The percentages of participants in risk category low, moderate or high at follow-up per baseline risk category			The differences between the percentages of participants from the various demographic groups and all participants								
		All	Age category (in January 2010)			Gender		Socioeconomic status			
Risk category at baseline	Risk category at follow-up	(%)	0–3	4–9	10–12	Boy	Girl	Low	Middle	High	Unknown
			n = 3,766	n = 16,945	n = 7,594	n = 14,451	n = 13,854	n = 9,147	n = 11,260	n = 7,526	n = 372
			(%)	(%)	(%)	(%)	(%)	(%)	(%)	(%)	(%)
	**High**	**38**	**+20**	**-6**	**+12**	**0**	**0**	**+4**	**-2**	**-6**	**+7**
**High**	Moderate	17	-1	+1	-2	0	0	0	+1	0	-7
(n = 5,505)	Low	45	-19	+5	-10	0	0	-4	+1	+6	+1
	High	25	+22	-4	+5	-1	+1	+4	-1	-4	+1
**Moderate**	**Moderate**	**18**	**+2**	**-0**	**0**	**0**	**0**	**+2**	**0**	**-2**	**-6**
(n = 3,650)	Low	57	-24	+4	-5	+1	-1	-5	+1	+6	+5
	High	14	+4	-3	+3	0	0	+3	0	-2	0
**Low**	Moderate	12	-1	0	+1	0	0	+1	0	-1	+2
(n = 19,150)	**Low**	**74**	**-4**	**+3**	**-5**	**0**	**0**	**-4**	**+1**	**+3**	**-2**
**The percentages of participants with a higher or lower risk category at follow-up**			**The differences between the percentages of participants from the various demographic groups and all participants**								
		All	Age category (in January 2010)			Gender		Socioeconomic status			
		(%)	0–3 (%)	4–9 (%)	10–12 (%)	Boy (%)	Girl (%)	Low (%)	Middle (%)	High (%)	Unknown (%)
Improved (lower risk category)		20	-13	+5	-4	0	0	+2	0	-2	-3
Deteriorated (higher risk category)		21	+7	-4	+5	0	0	+2	0	-2	+2

The changes in categorization were different for the various age groups and socioeconomic groups. Gender did not have any relevant effect. Participants who improved by moving to a lower risk category were more often from age group 4–9 (+5% compared to all) and socioeconomic status low (+2%). The youngest age group showed the least improvement (-13%). Participants who deteriorated by moving to a higher risk category were more often from the youngest (+7%) and eldest age groups (+5%), and socioeconomic status low (+2%).

## Discussion

This study showed that restorations in the past were the best predictor for future restorations. We categorized the number of restorations in the recent past into risk categories. The univariate results of the multilevel analysis (S2 Table) showed that the baseline risk category was the strongest predictor for the number of restorations during follow-up. The group of participants with zero restorations during the baseline period (2010–2011) got on average 0.81 new restorations, the group of participants with one restoration during baseline twice as many (1.61) and the group of children with two or more restorations during baseline even more than three times as many (2.65). Past restorations categorized into risk categories were also predictive for the percentage of participants with one or more new restorations during follow-up. For baseline risk category low this was 32%, for moderate 55%, and for high 70% of the participants. This is in line with earlier findings of Broadbent et al. [9] and Hummel et al. [10], which demonstrated that higher caries severity at baseline preceded higher caries increments.

The prediction model tended to overestimate the number of new restorations in individual cases. This was also reflected by the low positive predictive value for >0 restorations due to the large number of false positives. Using our model in individual cases could result in overtreatment and unnecessary costs for preventive interventions [11]. This finding supports the statement of Divaris (2016) that it is incorrect to apply population risk factors to individuals. Nonetheless, based on longitudinal observations, risk can be estimated for (subgroups in) populations in order to identify vulnerable groups and deploy extra resources [4]. Our classification model is helpful in identifying these groups.

Claimed restorations are only a proxy for caries activity. We assumed that restorations were indicated, and that this was because of caries, and not due to trauma or tooth wear. Claimed restorations are the result of the decisions of general dental practitioners (GDPs) to intervene restoratively. This is influenced by their opinions about good care and treatment policy for the primary dentition, the depth of a caries lesion, and the treatability of (young) children.

Claims data for other procedures were used as proxies for risk factors. We assumed that these procedures were individually indicated. That for example, the frequency of ROEs was higher due to the higher caries risk of a participant. However, some procedures, like sealants, might rather reflect the policy of a GDP than patient based indications. Work needs to be done to determine the impact of GDPs. Professional tooth cleaning was protective for future restorations. This was unexpected. During our study years there were no claims codes for oral hygiene instructions. These might have been claimed as tooth cleaning and indicate that attention for prevention had an effect. Almost 12% of the children in risk category low had tooth extractions during baseline. If those were indicated due to caries, these children were misclassified. However, the reasons for extractions were unknown, and we assumed that they were not related to caries in this group.

The overrepresentation of patients in JTVs is probably caused by a better recall system and a consistent use of the identification numbers for their DPs. Other DPs more often used multiple identification numbers in claims making it look like their patients had visited multiple DPs. Patients from JTVs are more often from socioeconomic status low. So, our estimates for the number of restorations may be overestimated as socioeconomic status low is associated with more restorations.

Our results apply to the 10% of the children that met our inclusion criteria. We do not know to what extent they apply to all children. The mean number of new restorations was comparable; 1.31 (SD 2.52) in all the children in the provided dataset versus 1.27 (SD 2.31) in our study population, but it is unknown whether the distribution of new restorations was similar.

The data we used in this study was obtained in 2016 and consisted of historical claims data. We believe that, despite the use of older data, there is no reason to assume that the results cannot be translated to or used in current practice. First of all, our data show practice patterns that are quite stable over the years. That is, over the last 15 years (since 2006) there have been changes in neither the oral healthcare insurance for children nor the remuneration system (fee-per-item). The same holds for practice guidelines, as over the last decade instructions for selfcare and maintenance of oral health remained the same. Hence we consider a bias due to a period or cohort effect unlikely. Nonetheless, the number of provided oral hygiene instructions has been increasing since 2012 and an increasing number of GDPs has been adopting a more non-operative caries treatment and prevention approach. Their aim is to stimulate long lasting better selfcare. In the future, this might result in less restorations, which will be reflected in a different distribution of risk categories in the population. By then, the prediction of the mean number of future restorations in the population can be corrected for this changed distribution of baseline risk categories.

Our model is of interest from a public oral health perspective. It provides a simple instrument for DPs to compare the number of restorations in their patient populations to reference figures. Policy makers can use our results for the population-based planning of oral healthcare services. Categorizing a population into risk categories and analyzing the provided care per risk category can be used as input for a risk-based capitation system. Finally, our model can be used as a quality measure, like changes in the distribution of risk categories. Harris et al. [12] considered the distribution of risk categories as one of the most useful outcome measures they tested, but the optimal period for reassessment needed to be determined yet. Our findings showed that participants could change risk categories over time and that 2 years is enough to detect changes in the distribution.

The model derivation and evaluation were performed in the same dataset. Although this was a very large dataset, validation is necessary. Caries risk assessment models are not automatically applicable to other populations. Especially not when they have other caries experiences and characteristics [1, 2]. It should be taken into account that the average caries experience in Dutch children is very low [13]. Further research needs to be done on the generalizability of our model to other populations.

## Conclusions

In this study, we developed and evaluated a classification model for dental caries risk in children and adolescents. This rested on categories of past caries experience derived from restoration claims from health insurance records. Like baseline caries experience was the single best predictor for future caries development in populations, one or more recent restorations were the single best predictor for future restorations. Our classification was also related to the number of future restorations in groups. Hence, this model can support planning and evaluation of oral healthcare services including a risk-based capitation system. Further research is needed to validate this model in other populations.

## Supporting information

S1 Table Derivation of the caries risk categories. Translation of a caries risk classification based on clinical findings into the classification model based on claims data.
(DOCX)

S2 Table Univariate results of the multilevel analysis. Univariate relationship between baseline variables and the number of restorations during three years of follow-up.
(DOCX)
