# Supplementary material for: The relationship between claimed restorations and future restorations in children and adolescents: An observational follow-up study on risk categories for dental caries
Source: PLoS One. 2021 Nov 12;16(11):e0259495. doi: 10.1371/journal.pone.0259495 (PMC8589182; doi:10.1371/journal.pone.0259495)
Supplement: S1 Table — Translation of a caries risk classification based on clinical findings into the classification model based on claims data. (DOCX) [file pone.0259495.s001.docx]

**S1 Table. Derivation of the caries risk categories.**

Translation of a caries risk classification based on clinical findings into the classification model based on claims data.

| **Description Mettes et al. 2010 ^1)^** | | **Modification Hummel et al.** | | |
| --- | --- | --- | --- | --- |
| **Risk category** | **Description** | **Risk category** | **Description** | **Explication** |
| **High** | Presence of (recurrent) active carious lesions, and increment of ≥2 new, progressing or filled lesions a year or ≥1 new lesion a year in subsequent years | **High** | Two or more new restorations in the last 2 years | Increment of ≥2 lesions in 1 year or ≥1 in subsequent years is translated into ≥2 new restorations in a period of 2 years |
| **Elevated** | Presence of active carious lesions, or increment of 1 new, progressing or filled lesion after a period of reduced or low-risk | **Moderate** | One new restoration in the last 2 years | Increment of 1 lesion is translated into 1 new restoration. A period of 2 years is added as it takes time for an active carious lesion to progress towards a stage needing restorative treatment. |
| **Reduced** | Previous disease experience, no active lesions or restorations due to caries in preceding 2 years or more | **Low** | No new restorations in the last 2 years | Mettes’ categories ‘reduced’ and ‘low’ were merged into 1 category ’low’ as we based our categorization on 2 ‘preceding’ years |
| **Low** | No caries experience |  |  |  |

^1)^ Mettes TG, van der Sanden WJ, van Eeten-Kruiskamp L, Mulder J, Wensing M, Grol RP, et al. Routine oral examination: clinical vignettes, a promising tool for continuing professional development? J Dent. 2010 May;38(5):377-86.
