# Supplementary material for: The relationship between claimed restorations and future restorations in children and adolescents: An observational follow-up study on risk categories for dental caries
Source: PLoS One. 2021 Nov 12;16(11):e0259495. doi: 10.1371/journal.pone.0259495 (PMC8589182; doi:10.1371/journal.pone.0259495)
Supplement: S2 Table — Univariate relationship between baseline variables and the number of restorations during three years of follow-up. (DOCX) [file pone.0259495.s002.docx]

**S2 Table. Univariate results of the multilevel analysis.**

Univariate relationship between baseline variables and the number of restorations during three years of follow-up.

| **Variable** | **rate ratio** | **p-value** |  | **95% confidence interval** | |
| --- | --- | --- | --- | --- | --- |
|  |  |  |  | **low** | **high** |
| Risk category high at baseline  Risk category moderate at baseline | 3.08  1.97 | <0.001  <0.001 |  | 2.94  1.86 | 3.24  2.09 |
| Age category 10-12 (January 2010)  Age category 4-9 | 1.09  0.75 | 0.02  <0.001 |  | 1.02  0.71 | 1.17  0.80 |
| Girls | 0.98 | 0.45 |  | 0.94 | 1.03 |
| Socioeconomic status high | 0.65 | <0.001 |  | 0.61 | 0.69 |
| Socioeconomic status middle | 0.78 | <0.001 |  | 0.74 | 0.82 |
| Socioeconomic status unknown | 0.76 | 0.004 |  | 0.63 | 0.91 |
| Patient in JTV ^a^ | 1.10 | 0.53 |  | 0.82 | 1.48 |
| More than 4 routine oral health examinations  during baseline | 1.42 | <0.001 |  | 1.21 | 1.66 |
| Intra-oral radiographs during baseline | 1.37 | <0.001 |  | 1.31 | 1.45 |
| Professional fluoride applications during baseline | 0.91 | <0.001 |  | 0.86 | 0.95 |
| Professional toothcleanings during baseline | 0.96 | 0.10 |  | 0.91 | 1.01 |
| Dietary advice during baseline | 1.72 | 0.01 |  | 1.15 | 2.58 |
| Plaque scores during baseline | 1.23 | <0.001 |  | 1.14 | 1.32 |
| Sealants during baseline | 1.10 | <0.001 |  | 1.05 | 1.15 |
| Tooth extractions during baseline | 1.24 | <0.001 |  | 1.17 | 1.31 |
| Endodontic treatments during baseline | 2.77 | <0.001 |  | 2.07 | 3.71 |
| Endodontic treatments in primary teeth during baseline | 1.75 | <0.001 |  | 1.31 | 2.32 |
| ^a^ JTV is a center specialized in pediatric oral healthcare | | | | | |
